# Online Metaphor Comprehension in Adults with Autism Spectrum Disorders: An Eye Tracking Study

**DOI:** 10.1007/s10803-024-06562-5

**Published:** 2024-09-21

**Authors:** Aimee O’Shea, Rita Cersosimo, Paul E. Engelhardt

**Affiliations:** 1 School of Psychology, University of East Anglia, Norwich Research Park, Norwich, UK; 2 University of Genoa, Genoa, Italy

**Keywords:** Autism spectrum disorders, Metaphor processing, Language ability, Pragmatics, Social communication

## Abstract

The aim of this study was to investigate novel metaphor comprehension in adults with autism spectrum disorder (ASD). Previous literature is conflicting about whether individuals with ASD have impairment in this particular type of figurative language. Participants in the study completed a visual world paradigm eye-tracking task, which involved selecting an interpretation of an auditorily presented sentence (i.e. a picture-sentence matching task), where images corresponded to literal and metaphorical interpretations. Thus, the study also investigated online processing, via reaction times and eye movements. Forty adults participated in the study (18 with ASD and 22 typically-developing controls). Each participant completed the AQ questionnaire and had their vocabulary assessed. Results showed that participants with ASD comprehended metaphorical utterances with the same accuracy as controls. However, they had significantly slower reaction times, and specifically, were approximately 800 ms slower. Analysis of eye movements revealed that participants with ASD showed significantly longer fixation times on both the target and distractor image, the latter of which suggests difficulty overcoming the literal interpretation. Consistent with some prior studies, we showed that adults with ASD are not impaired in novel metaphor comprehension, but they were clearly less efficient. Verbal abilities did not significantly relate to performance. Finally, our online processing measure (eye tracking) provided us with insights into the nature of the ASD inefficiency (i.e. a literality bias).

**Supplementary Information:**

The online version contains supplementary material available at 10.1007/s10803-024-06562-5.

Metaphor comprehension is commonly considered challenging for individuals on the Autism Spectrum. The latest edition of the Diagnostic and Statistical Manual of the American Psychiatric Association (DSM-5; 2013) includes difficulties in understanding non-literal and ambiguous language meanings, such as metaphors, as key criteria for ASD. However, recent findings cast doubt on the notion that individuals with ASD encounter metaphor processing difficulties, beyond those attributed to broader language impairments (Brock et al., 2008; Gernsbacher & Pripas-Kapit, 2012; Norbury, 2005).

The finding that metaphors are not understood (or only partially understood) by people with ASD was first proposed in seminal work by Happé (1993), who linked difficulties in understanding figurative language to weaknesses in Theory of Mind (ToM), frequently found in ASD (Baron-Cohen et al., 2001). ToM, defined as the ability to attribute mental states to oneself and others (Frank, 2018), is essential for interpreting beliefs, intentions, thoughts, and emotions, thereby directly influencing communicative pragmatics. However, the precise role of ToM in metaphor comprehension remains a subject of much debate (Bosco, 2018; Gernsbacher & Pripas-Kapit, 2012). Happé (1993) proposed that metaphors, unlike similes, necessitate the recipient to discern the speaker’s intention as the meaning conveyed is non-literal. To test this hypothesis, Happé employed a sentence completion task including synonyms, similes, and metaphors, differentiating participants based on impairments in first-order, second-order, or both orders of ToM. First-order ToM pertains to inferring another individual’s mental states, whereas second-order ToM involves deducing another individual’s mental states concerning a third party (Duval et al., 2011). The group with impairments in both orders of ToM exhibited lower scores (only) in the metaphorical condition, while the other two groups did not manifest significant differences from each other.

Several subsequent research studies contributed to the idea that people with ASD have issues with figurative language comprehension (e.g., Dennis et al., 2001; Kaland et al., 2002; MacKay & Shaw, 2004). Lower accuracy in metaphor tasks was confirmed by recent meta-analyses (Kalandadze et al., 2018; Morsanyi et al., 2020), which found medium-to-large group differences (Hedges’ g was respectively 0.63 and 0.76). However, both meta-analyses caution against drawing firm conclusions from these results because of heterogeneity in the studies. The main reasons for variability are (i) participants’ individual differences in linguistic abilities, and (ii) the format of the tasks used to assess metaphor comprehension.

Interestingly, a number of research studies found that group differences are no longer significant when the ASD and control groups were matched on verbal ability (e.g., Brock et al., 2008; Gernsbacher & Pripas-Kapit, 2012; Geurts et al., 2020; Kalandadze et al., 2018; Norbury, 2005). Metaphors require individuals to perceive similarities between two terms typically regarded as distinct, often involving features that are not the most salient in either term (Giora et al., 2012). Therefore, a comprehender must possess enough world knowledge and sufficiently broad semantic representations to grasp the intended comparison (Norbury, 2004). Morsanyi et al. (2020) observed a notable impact of verbal intelligence, where distinctions in metaphor processing between ASD and control groups were reduced or absent among participants with higher verbal skills. Notably, approximately 60% of the variance in effect sizes across studies could be attributed to variance in participants’ verbal abilities.

Norbury (2005) was among the first to highlight the significance of language skills in metaphor processing in ASD. Contrary to the assumption that ToM deficits solely account for difficulties in understanding metaphorical language, Norbury observed that children with language impairments encounter challenges in comprehending metaphors, despite maintaining relatively intact ToM abilities (Highnam et al., 1999; Leslie & Frith, 1988; Rinaldi et al., 2006; Shields et al., 1996; Ziatas et al., 1998). In her investigation, Norbury found that only children with language impairment, with or without concurrent ASD features, displayed difficulties in metaphorical tasks. Moreover, possessing first-order ToM skills did not guarantee metaphor comprehension. Instead, Norbury highlighted semantic abilities as a more robust predictor of performance on metaphor tasks. Consequently, she underscored the centrality of semantic knowledge, suggesting that ToM skills facilitate metaphor understanding by enriching contextual representations.

Language skills are extremely variable across the autism spectrum. While many individuals with ASD develop language skills within the typical trajectory observed in typically-developing individuals (Friedman & Sterling, 2019; Kjelgaard & Tager-Flusberg, 2001), a substantial proportion, estimated between 25 and 30%, remain non-verbal or possess only minimal verbal abilities (Pickles et al., 2014; Tager-Flusberg & Kasari, 2013). Studies investigating metaphor comprehension predominantly involve people with average functional language skills. Contrary to the assumption that deficits in figurative language comprehension are universal among individuals with ASD (Gernsbacher & Pripas-Kapit, 2012), some researchers suggest that these challenges may not be specific to ASD and could instead be linked to individual structural language abilities, including vocabulary and syntax (Norbury, 2004, 2005; Whyte et al., 2014). In another study, Norbury (2004) reported that children and adolescents with ASD did not exhibit impairments in figurative language comprehension when their vocabulary and syntactic skills fell within the normal range. This finding was corroborated by a meta-analysis conducted by Kalandadze et al. (2018), which identified language ability as a significant contributor to the variability in figurative language comprehension across studies. Specifically, when comparing the performance of individuals with ASD and typically-developing peers on core language assessments, the effect size was found to be small and not significant (Hedges’ g =  − 0.06). Therefore, the overall challenges encountered in figurative language comprehension appear to be more directly associated with core language skills, rather than ToM.

There are also studies, which found no significant difference between ASD and typically-developing individuals in the comprehension of metaphors (e.g., Chouinard & Cummine, 2016; Hermann et al., 2013; Kasirer & Mashal, 2016; Olofson et al., 2014). Hermann et al. (2013) and Chouinard and Cummine (2016) used the Metaphor Interference Effect paradigm to investigate the initial stages of metaphor comprehension. This semantic-judgment task was useful to discern the time where a metaphorical meaning was generated from the time where unintended meanings were suppressed. In fact, the generation of metaphorical meaning may occur independently of understanding the metaphor in conversation, which requires subsequent steps of inhibiting the literal meaning and integrating the metaphorical utterance within discourse and social contexts. Their findings suggested that individuals with ASD correctly generate the metaphorical meaning, but potential difficulties arise in suppressing irrelevant literal features.

Another factor that might influence metaphor comprehension is metaphor novelty. In contrast to familiar metaphors, the meaning of novel metaphors is not immediately accessible and requires on-the-spot computation. Previous studies in the literature have consistently indicated that novel metaphors demand greater cognitive effort (e.g., Bowdle & Gentner, 2005). For example, Arzouan et al. (2007) utilized Event-Related Potentials (ERPs) to examine the impact of novelty on metaphor processing. Results from their semantic-judgment task revealed a linear increase in the N400 effect, progressing from literally related words to conventional metaphors, novel metaphors, and semantically unrelated pairs.

However, the few available studies on metaphor comprehension in ASD that have investigated the distinction between novel and familiar metaphors have not consistently supported the notion of increased difficulty with novel metaphors (Hermann et al., 2013; Kasirer & Mashal, 2014; Mashal & Kasirer, 2011; Melogno et al., 2012). For example, in Kasirer and Mashal (2016), children with ASD showed lower comprehension of conventional metaphors compared to controls, but no significant differences were observed in the comprehension of novel metaphors. The authors attributed this finding to pragmatic difficulties commonly associated with ASD (Tager-Flusberg, 2003), as familiar expressions rely on pre-existing knowledge (Pouscoulous, 2014) and shared understanding between speakers (Ritchie, 2004).

Metaphor comprehension in ASD has also been examined by analyzing reaction times to metaphorical stimuli. Gold et al. (2010) reported longer response times and greater N400 amplitudes in a group with ASD compared to typically-developing individuals, despite similar accuracy rates. They suggested that this slower processing may be due to difficulties in suppressing irrelevant semantic components, aligning with findings from Metaphor Interference Effect studies (Chouinard & Cummine, 2016; Hermann et al., 2013). Morsanyi et al. (2020) considered reaction time results related to metaphor processing in their meta-analysis. Drawing from the findings of four studies that reported both accuracy and reaction times for metaphor processing (Chahboun et al., 2017; Gold et al., 2010; Gold & Faust, 2010; Morsanyi & Stamenkovic, 2021), they observed a general advantage for typically-developing individuals where the reaction time was lower (Hedges’ g = 0.74). Thus, people with ASD are not necessarily less accurate in metaphor comprehension, but the few studies which assessed online performance (i.e., reaction times) seem to suggest slower processing.

Another source of heterogeneity among studies investigating metaphor comprehension in ASD is the variety of tasks employed. In a meta-analysis, Kalandadze et al. (2018) observed that the method of assessing metaphor comprehension can yield varying outcomes. For example, tasks that necessitate individuals to explain the meaning of a metaphor may pose particular challenges for people with ASD due to difficulties with expressive language (Kwok et al., 2015). Another meta-analysis (Morsanyi et al., 2020) corroborated this finding, emphasizing that studies that presented more substantial and consistent group disparities, typically used tasks demanding more complex responses. These tasks included verbalizing metaphors depicted in images (Tzuriel & Groman, 2017), explaining a metaphorical meaning (Borkowska, 2015; de Villiers et al., 2011; Landa & Goldberg, 2005), or drawing inferences based on ambiguous metaphorical expressions (Minshew et al., 1995). The most common assessment format and the one that is less likely to show significant group differences is multiple choice, which provides participants with clear interpretation options. Multiple choice, therefore, falls more on the simpler end of the continuum of task difficulty.

In summary, while individuals with ASD often face more pronounced challenges in metaphor comprehension, attributing issues in figurative language as a hallmark of ASD remains a topic of contention. It is crucial to acknowledge that multiple factors, including language skills and task formats, may contribute to group differences. Therefore, further research is needed to shed light on (1) whether differences exist and if they do, (2) what are the underlying causes of the difficulties.

## Current Study

The current study focused on providing further evidence about how adults with ASD understand metaphors through the use of eye-tracking data, combined with response time and comprehension accuracy. There are several prior eye tracking studies of metaphor comprehension and those involved eye movements in reading, but did not assess individuals with ASD (e.g. Ashby et al., 2018; Columbus et al., 2015; Ronderos et al., 2021; cf. Coulson et al., 2015). To date, there has been only one eye tracking study to explore metaphor comprehension in ASD (Vulchanova et al., 2019), and that study recorded eye movements, while participants viewed pictures on the computer screen. As mentioned previously, the majority of previous research on metaphor comprehension in ASD only included behavioral assessment, and only a handful report online measures of processing (e.g. reaction time, eye movements, or neuroimaging). As suggested by Kalandadze et al. (2018), “more high-quality studies on metaphor comprehension in ASD are needed combining offline and online comprehension methods widely used in psycholinguistic research” (p. 1447). Online processing tasks measure implicit processing and provide more fine-grained insights into how metaphors are understood by people with ASD.

Additionally, there is little data on adults with ASD in comparison to research conducted on children and adolescents (cf. Vulchanova et al., 2019), and the findings thus far appear to be inconclusive. In their examination of figurative language, Saban-Bezalel and Mashal (2015) determined that challenges in metaphor processing among individuals with ASD are more evident during childhood and adolescence, but may not persist into adulthood, although this outcome is contingent upon the specific type of metaphor task and the manner in which responses are elicited (see also Chahboun et al., 2017; Vluchanova et al., 2019). Saban-Bezalel and Mashal analyzed a study conducted by Kasirer and Mashal (2014), where adults with ASD and controls underwent a metaphor comprehension multiple-choice test and no differences were found. By contrast, a review conducted by Vulchanova et al. (2015) concluded that disparities in metaphor processing persist among high-functioning individuals with ASD due to challenges related to integrating information. The authors highlighted the significance of various cognitive and linguistic skills in metaphor comprehension, emphasizing that deficits in any of these areas can impede the processing of figurative language.

Since metaphor processing relies heavily on semantic skills, our study is aimed at providing further insights on how adults with ASD understand metaphors by assessing vocabulary skills (one component of verbal ability) together with metaphor comprehension. Based on the existing literature, we hypothesized that no substantial differences in accuracy would emerge if the two groups do not differ on vocabulary skills, as Kalandadze et al. (2018) and Morsanyi et al. (2020) point out in their meta-analyses. Still, it is possible that slowness in computing the correct meaning would arise, as suggested in the few studies that took reaction times into consideration (e.g. Chahboun et al., 2017; Gold et al., 2010; Gold & Faust, 2010; Morsanyi & Stamenkovic, 2021).

**Fig. 1:**
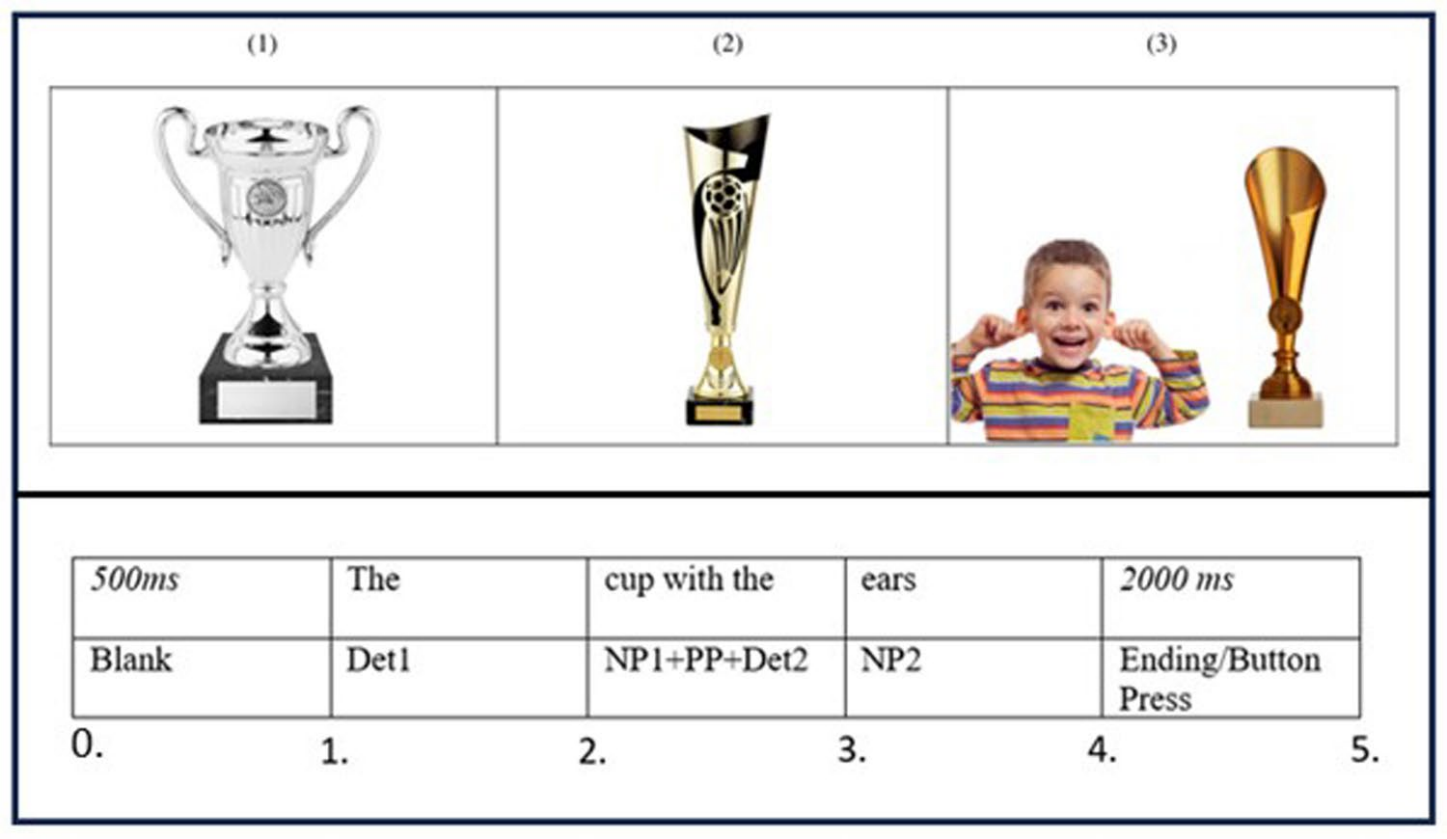
Top panel shows example visual array: the metaphorical utterance was “the cup with the ears” and the literal utterance was “the cup with the handles”. Image (1) is the target picture, image (2) is the irrelevant picture, and image (3) is the distractor picture. Bottom panel shows the key time points for dividing the sentence into critical time periods

Eye-tracking data, particularly the pattern of fixations to different visual representations, allowed us to clarify how the process of understanding a metaphor unfolds in adults with ASD. Longer fixations on the picture representing the literal meaning (i.e., the distractor image, see Materials section, Fig. 1) would indicate a possible “literality bias” already mentioned by some authors (see Rossetti et al., 2018) or, alternatively, the difficulty in rejecting irrelevant meaning that emerged in the studies that used the Metaphor Interference Effect in ASD (Chouinard & Cummine, 2016; Hermann et al., 2013). Accuracy data would allow us to discriminate between the two: lower accuracy due to a high number of literal interpretations is more likely to be attributed to the former, while higher accuracy to the latter.

In our task, we specifically used novel metaphors to limit potential confounding factors that may arise when interpreting more conventionalized metaphors, which rely on shared knowledge between speakers and well-developed pragmatic skills (Kasirer & Mashal, 2016; Vluchanova et al., 2019). In contrast, the meaning of novel metaphors is not stored in the mental lexicon, and their interpretation does not hinge on prior knowledge, but rather on immediate meaning computation and semantic associations between the two metaphor terms. Novel metaphors are innovative and context-specific and require pragmatic inference that involves adjusting meaning to the specific context (Recanati, 2004; Sperber & Wilson, 2012; Wilson & Carston, 2006).

## Methods

### Participants

Forty-one undergraduate students participated in this study.^1^ There were 18 with ASD and 22 typically-developing adults were tested as control participants (see Table 1). Both groups were recruited from the University of East Anglia (UEA). All participants with autism verified that they had diagnostic assessments for autism in the past, and were currently on the disability register at UEA. The mean age of ASD diagnosis was 14.38 years (*SD* = 5.95). Twelve ASD participants obtained their diagnosis via the NHS, and five were obtained from a private psychiatric practice or autism specialist service. All were native speakers of English with normal or corrected-to-normal vision. Participants were compensated for their time either with participation credits or with a £7 Amazon voucher. The study was approved by the School of Psychology Research Ethics Committee at the University of East Anglia (UK). Informed consent was obtained from all participants before carrying out the study and all were debriefed at the end of the study.

**Table 1:** Means for demographic variables, vocabulary, and ASD screening measures

	ASD (18)	Contro l(22)	Significance
Variable	Mean (SD)	Mean (SD)	
Age	19.89 (1.81)	19.91 (2.52)	*t*(38) = − 0.28, *p* = *0.98*
Gender (% male)	50.0	27.3	*t*(36) = 2.08, *p* < 0.05
AQ SS	6.50 (1.82)	2.41 (1.79)	*t*(38) = 7.13, *p* < 0.001
AQ AS	8.89 (1.23)	5.32 (2.06)	*t*(38) = 6.47, *p* < 0.001
AQ AD	7.00 (2.17)	5.73 (2.55)	*t*(38) = 1.68, *p* = 0.10
AQ COM	7.78 (2.02)	3.05 (2.40)	*t*(38) = 6.66, *p* < 0.001
AQ IMG	5.06 (2.24)	2.23 (1.60)	*t*(38) = 4.66, *p* < 0.001
AQ TOTAL	35.22 (7.12)	18.73 (6.16)	*t*(38) = 7.86, *p* < 0.001
PPVT	41.56 (7.91)	40.10 (8.95)	*t*(38) = 0.54, *p* = 0.59

Two participants with ASD reported non-binary gender, and one control reported “other” gender. These participants were not included in any gender analysis.

### Materials

All participants were tested individually before the eye-tracking task. The standardised procedures of administration for each test were followed as described in the test manuals.

*Peabody Picture Vocabulary Test-4 (PPVT-4).* The PPVT-4 (Dunn & Dunn, 2007) is a tool to assesses receptive vocabulary. The researcher aurally presented a target word and participants were asked to choose the image which best illustrated the meaning between four. The reliability range for Form A (the one used in this study) is reported to be from 0.89 to 0.97.

*Autism-Spectrum Quotient.* The AQ is a self-report measure of autistic traits (Baron-Cohen et al., 2001), consisting of 50 items assessing ASD symptomology in five areas (social skills, attention switching, attention to detail, communication, and imagination). Answers are given on a four-point Likert scale with the options ‘Definitely Agree’, ‘Slightly Agree’, ‘Slightly Disagree’ and ‘Definitely Disagree’. Scores on the AQ are summed and can range from 0 to 50, with a higher score indicating that the individual possesses a higher level of autistic traits. For the purpose of the current study, subscales of the AQ were also summed. Descriptive statistics for total AQ score as well as subscale scores across both the ASD group and the control group can be seen in Table 1.

#### Metaphor Comprehension Task

The metaphor comprehension task utilized a version of the Visual World Paradigm (Tanenhaus et al., 1995) and stimuli were a combination of a visual array consisting of three pictures and an auditorily presented sentence. Stimuli for this task were adapted from Pouscoulous and Tomasello (2020) and Di Paola et al. (2018). Novel metaphors were created to be suitable for adult participants. Twenty pairs of novel metaphors were constructed. They were in a syntactic structure in the form [The X with the Y], where the qualifier (Y) was figurative in the metaphor condition. All sentences were similar in length. Nouns used in the (X) and (Y) positions were frequent and concrete. To check for the words concreteness and frequency, we used ratings from Brysbaert et al. (2014) and van Heuven et al. (2014). For each trial, a target picture and two control pictures were sourced: (1) the target picture showed the target object referred to either metaphorically or literally (e.g., a cup with handles for *The cup with the ears/handles*), (2) the irrelevant picture illustrated the metaphor target without the relevant property (e.g., a cup without handles), and (3) the distractor was a literal competitor, showing both target and vehicle (e.g., a cup and a boy pointing at his ears) (see Fig. 1).

We defined five key time points for each trial (see Fig. 1, bottom panel). Based on the key time points, we analysed two critical time windows. The first time window (Region 1) was from the onset of noun phrase 1 (NP1) to the onset of noun phrase 2 (NP2) (time point 2 to time point 3). The second time window (Region 2) was from the onset of NP2 to when the participant made a button response (time point 3 to time point 5).^2^

Metaphors were normed on a 7-points Likert scale for their familiarity, aptness, and conventionality following the same procedure, as in Dulcinati et al. (2014). Target pictures were normed for their suitability to the sentence (i.e., *How suitable is this image to represent the sentence?*). A total of 120 native English speakers, recruited through Prolific (www.prolific.co), took part in the norming task and were paid for their time, with groups of 30 unique participants assigned to each survey. Three sentences were considered as outliers and removed from the study. Two sentences showed high familiarity or conventionality ratings (between 4 and 7 points). One showed a low picture suitability (less than 3 points). All sentences were considered apt metaphors (i.e., are perceived as providing an accurate description of the topic). One sentence was taken as a practice item. A total of 16 sentences rated as apt novel metaphors were included in the study together with their 16 literal corresponding expressions and 32 fillers. (Half of the fillers were idioms, and half were controls in which the sentence referred to only one picture.) Materials and norming results are available on the Open Science Framework online data repository (https://osf.io/39bxk/).

#### Apparatus

Eye movements were recorded with an SR Research Ltd. EyeLink 1000 eye-tracker, which records the position of the reader’s eye every millisecond. Head movements were minimised with a chin rest. Eye movements were recorded from the right eye. Experiment Builder was used to program the experiment, and Data Viewer was used to extract the interest area reports for eye movements and message reports for button presses. The sentences were auditorily presented through a computer speaker.

#### Design and Procedure

The design was a 2 × 2 (Sentence Type × Group) mixed model, in which sentence type (literal and metaphorical) was within subject and group (ASD and typically-developing control) was between subject. Participants completed two practice trials, 32 experimental trials, and 36 fillers. Trials were presented in a random order for each participant. Critical trials were rotated in a Latin Square design, resulting in six lists of stimuli. Each critical utterance had a literal utterance counterpart, and target images were rotated through the different possible positions in the arrays.

Before the start, details of the tasks were given to the participants. The researcher answered any questions, if required. All participants gave written informed consent. Participants were first asked to fill out a demographic questionnaire and the AQ (Baron-Cohen et al., 2001), followed by the PPVT-4 (Dunn & Dunn, 2007). The researcher then administered the talking portions of the ADOS-2 (Lord et al., 2012). These preliminary measures took approximately 20 min.^3^

Participants were then guided to the experimental task, where they were required to sit at the eye tracker and respond to on-screen instructions using the keyboard. At the beginning of each trial, a message appeared asking the participant to press a button when they were ready to continue. After the participant pressed the button, they were required to fixate a drift-correction dot, which appeared in the centre of the screen. The experimenter then initiated the trial. Participants heard the sentences, while simultaneously being presented with three pictures on the screen. There were two practice trials. If the participant was ready and had no more questions, they proceeded to the critical trials.

For each trial, the audio file started 500 ms after the pictured appeared. There was a 2000 ms time window following the sentence in which participants needed to make their choice about which picture they thought best fit the sentence. They were asked to press ‘1’ if they wanted to choose the left picture, ‘2’ for the centre picture, and ‘3’ for the picture on the right. The eye-tracking testing session for each participant lasted approximately 5 min. To avoid any bias, participants were not informed of the inclusion of figurative language among the sentences. During the debrief, the aim of the experiment was explained in detail, and participants were compensated for their time before leaving. The experimental study took approximately 30 min to complete.

## Results

Outliers were defined by examining standardized scores and histogram plots. We use a threshold of 3.0 SDs from the mean. There were two datapoints exceeding this threshold for reaction times for one participant. We ran all analyses with this participant removed from the dataset, and the main effects and interactions fully replicated. Thus, we retained the outlier in the dataset (further information about the outlier can be found in the Supplementary Materials, Section D). The results section is organized in the following order: comprehension accuracy, reaction time for all trials, reaction time for correct and incorrect trials, and eye movements. Eye movement analyses focused on two interest periods (see Fig. 1). The eye movement dependent measure was summed fixation times (dwell time) on each picture in the array during critical interest periods. In the remainder of the paper, we use dwell time and fixations, interchangeably. All analyses used mixed model ANOVAs.

### Comprehension Accuracy

**Fig. 2:**
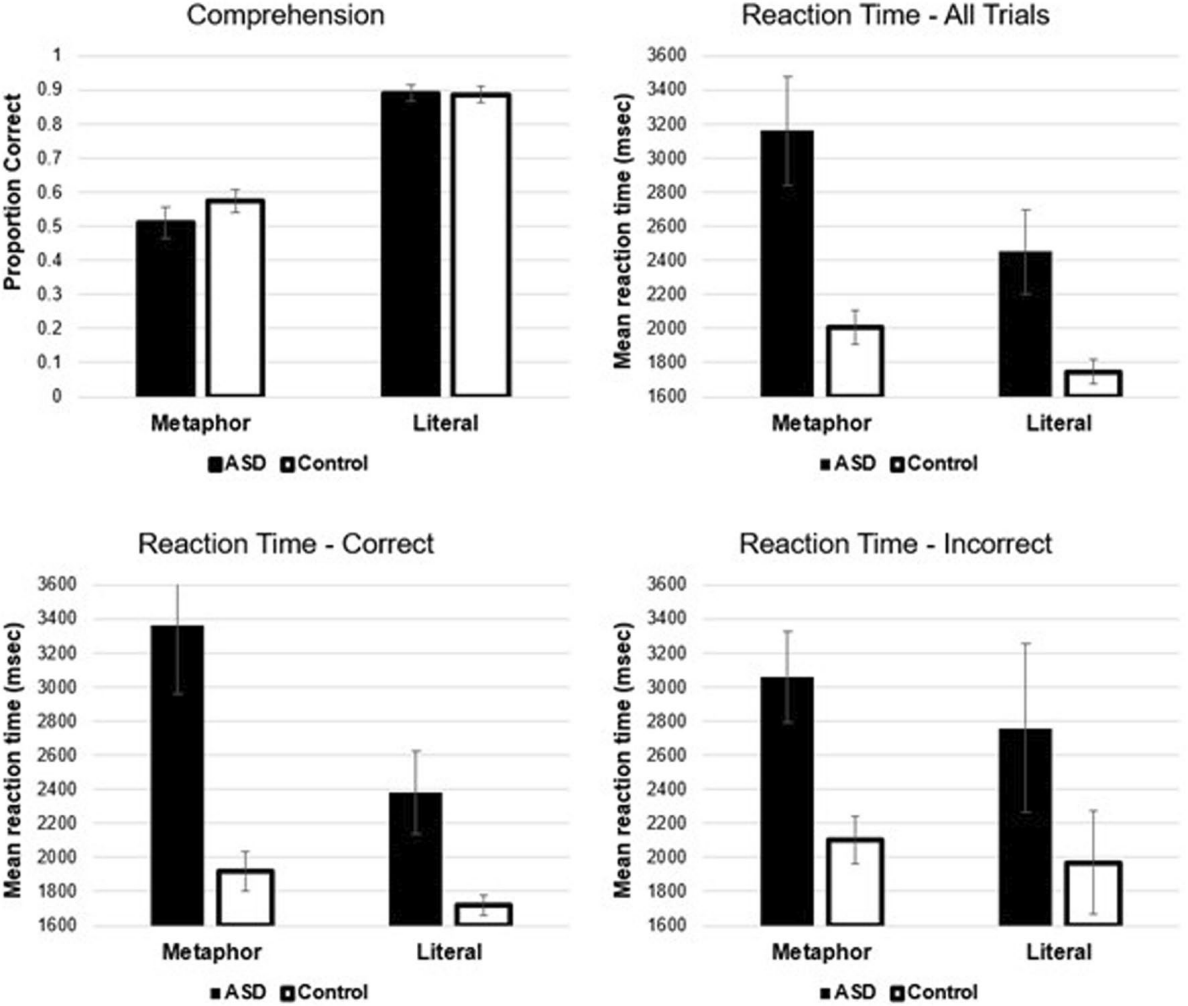
Upper left panel shows mean comprehension accuracy. Upper right panel shows mean reaction time for all trials. Bottom left panel shows mean reaction time for correct trials. Lower right panel shows the reaction time for incorrect trials. Note that there were very few literal trials that were incorrect. Error bars show the standard error of the mean

For comprehension accuracy, results showed a significant main effect of sentence type *F*(1,38) = 91.50, *p* < 0.001, *η*^*2*^ = 0.71, in which literal sentences had higher comprehension than did metaphor sentences (see Fig. 2). The main effect of group *F*(1,38) = 0.98, *p* = 0.33, *η*^*2*^ = 0.03 and the interaction *F*(1,38) = 0.98, *p* = 0.33, *η*^*2*^ = 0.03 were not significant.^4^

### Reaction Time—All Trials

Reaction times were computed from the onset of NP2 (time point 3) to when participants made the button press (see Fig. 1). For reaction time, results for all trials showed significant main effects of sentence type *F*(1,38) = 42.15, *p* < 0.001, *η*^*2*^ = 0.53, in which literal sentences were processed more quickly compared to metaphor sentences, and group *F*(1,38) = 12.22, *p* = 0.001, *η*^*2*^ = 0.24, where the control group had shorter reaction times than did the ASD group (see Fig. 2). The interaction was also significant *F*(1,38) = 9.01, *p* < 0.01, *η*^*2*^ = 0.19. Paired comparisons showed significant differences between literal and metaphor sentences for both groups: ASD *t*(17) =  − 4.96, *p* < 0.001, Cohen’s *D* =  − 1.17, and control *t*(21) =  − 3.83, *p* < 0.001, Cohen’s *D* =  − 0.82. The comparison of ASD vs. control showed significant differences for both metaphor trials *t*(38) = 3.72, *p* < 0.001, Cohen’s *D* = 1.18 and literal trials *t*(38) = 2.97, *p* < 0.01, Cohen’s *D* = 0.95. Thus, all paired comparisons were significant, but the interaction was based primarily on the elevated reaction times for metaphor trials in individuals with ASD.

### Reaction Time—Correct Trials

Reaction times for correct trials confirmed significant main effects of sentence type *F*(1,38) = 24.55, *p* < 0.001, *η*^*2*^ = 0.39 and group *F*(1,38) = 12.68, *p* = 0.001, *η*^*2*^ = 0.25, and a significant interaction between variables *F*(1,38) = 10.88, *p* < 0.01, *η*^*2*^ = 0 0.22 (see Fig. 2). Note that Fig. 2 also shows reaction times for incorrect trials. We did not analyse these trials statistically, but have included them for the purposes of comparison.^5^ Paired comparisons showed significant group differences for metaphor trials *t*(38) = 3.73, *p* < 0.001, Cohen’s *D* = 1.19 and for literal trials *t*(38) = 2.86, *p* < 0.01, Cohen’s *D* = 0.91. The comparison of metaphor and literal were significant for ASD *t*(17) =  − 4.09, *p* < 0.001, Cohen’s *D* =  − 0.96 and for controls *t*(21) =  − 2.22, *p* < 0.05, Cohen’s *D* =  − 0.47. Thus, again, the interaction was primarily driven by the elevated reaction times in metaphor trials in individuals with ASD.

### Metaphor Processing—Cost Analysis

There were significant differences for both literal and metaphorical trials in terms of reaction times between groups. As one final analysis of reaction time, we computed a difference score in which we subtracted the mean literal reaction time (correct trials) from the mean metaphor reaction time (correct trials). This difference score provides the metaphor processing costs, while taking into account literal (base-line) processing time. The difference score mean for ASD was 985 ms and for controls was 198 ms. This was a statistically significant difference *t*(38) = 3.30, *p* < 0.01, Cohen’s *D* = 1.05. Thus, participants with ASD took approximately 800 ms longer to process novel metaphors compared to controls.

### Eye Movements

For the eye movement analysis, we analysed group, sentence type, and picture type (target vs. distractor), which results in 2 × 2 × 2 mixed design. Picture type and sentence type were within subject and group was between subjects. Note that we did not include the irrelevant picture in statistical analyses, but did include it in the figures for comparison purposes. The main analyses focused on eye movements for correct trials. However, we also considered eye movement results for incorrect metaphor trials. (Incorrect responses were uncommon in literal trials.)

**Fig. 3:**
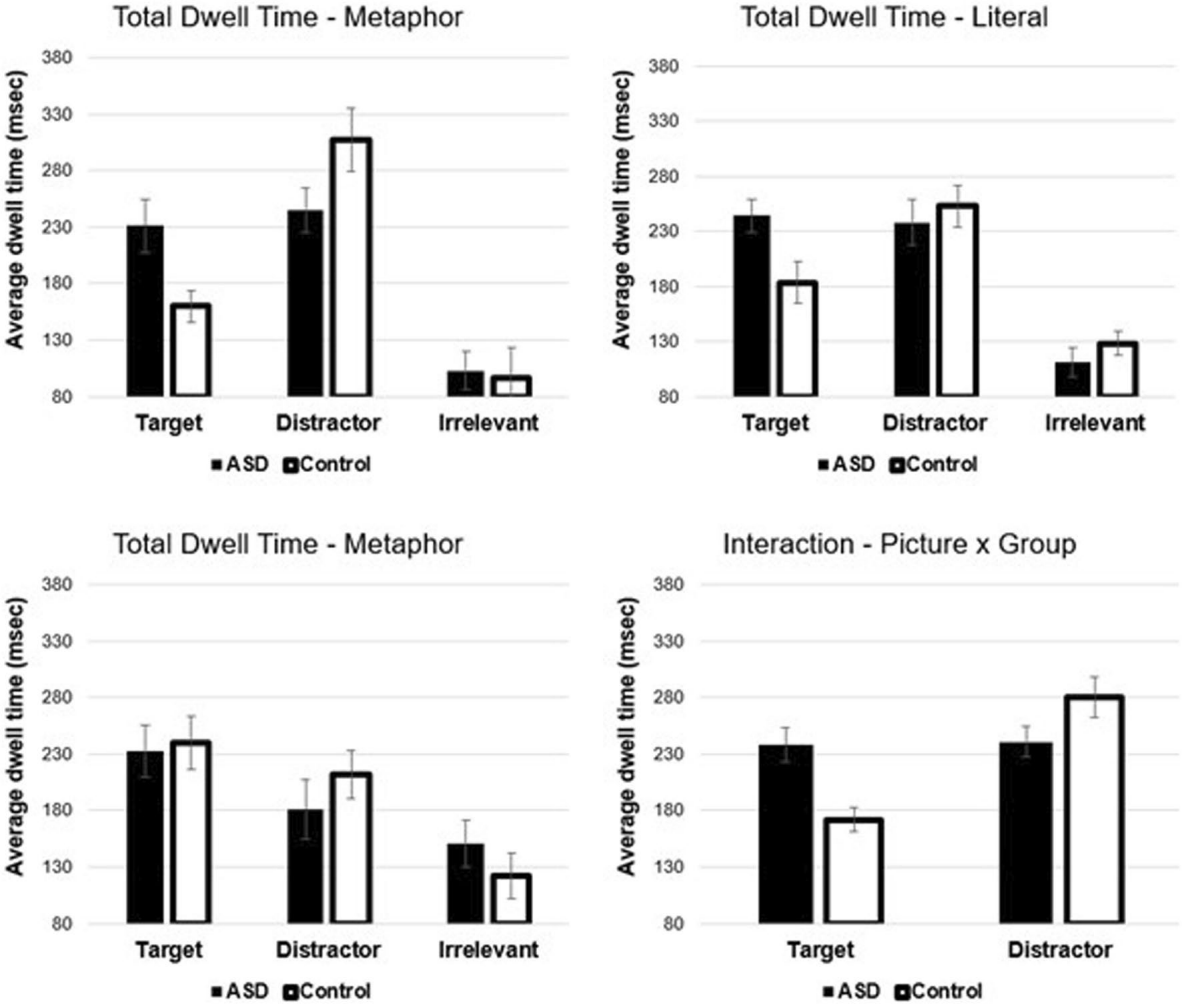
Mean fixation times. Upper panels show mean dwell times for the correct trials (left metaphorical and right literal). Lower left panel shows the mean dwell times for metaphor incorrect trials. Lower right panel shows the means for the picture by group interaction. Error bars show the standard error of the mean

*Region 1.* In the first time window (onset of NP1 to onset of NP2), there was a significant main effect of picture type *F*(1,38) = 11.97, *p* < 0.001, *η*^*2*^ = 0.24 (see Fig. 3). The distractor was viewed for longer compared to the target. There was also a significant interaction between picture type and group *F*(1,38) = 10.54, *p* < 0.01, *η*^*2*^ = 0.22 (see Fig. 3). Paired comparisons showed significant differences between ASD and controls for target fixations *t*(38) = 3.61, *p* < 0.001, Cohen’s *D* = 1.15 but not for distractor fixations *t*(38) =  − 1.67, *p* = 0.10, Cohen’s *D* =  − 0.53. There was also a significant difference comparing target to distractor for controls *t*(21) =  − 4.95, *p* < 0.001, Cohen’s *D* =  − 1.06, but not for the ASD group *t*(17) =  − 0.15, *p* = 0.89, Cohen’s *D* =  − 0.03.

Thus, the interaction was due to the fact that control participants spent more time fixating the distractor and less time fixating the target. In contrast, the ASD group showed equal fixation of the target and distractor, and significantly more target fixations, compared to the control group. None of the other main effects or interactions were significant. There were also no significant differences in metaphor incorrect trials (all *p’s* > 0.15) (see Fig. 3).

**Fig. 4:**
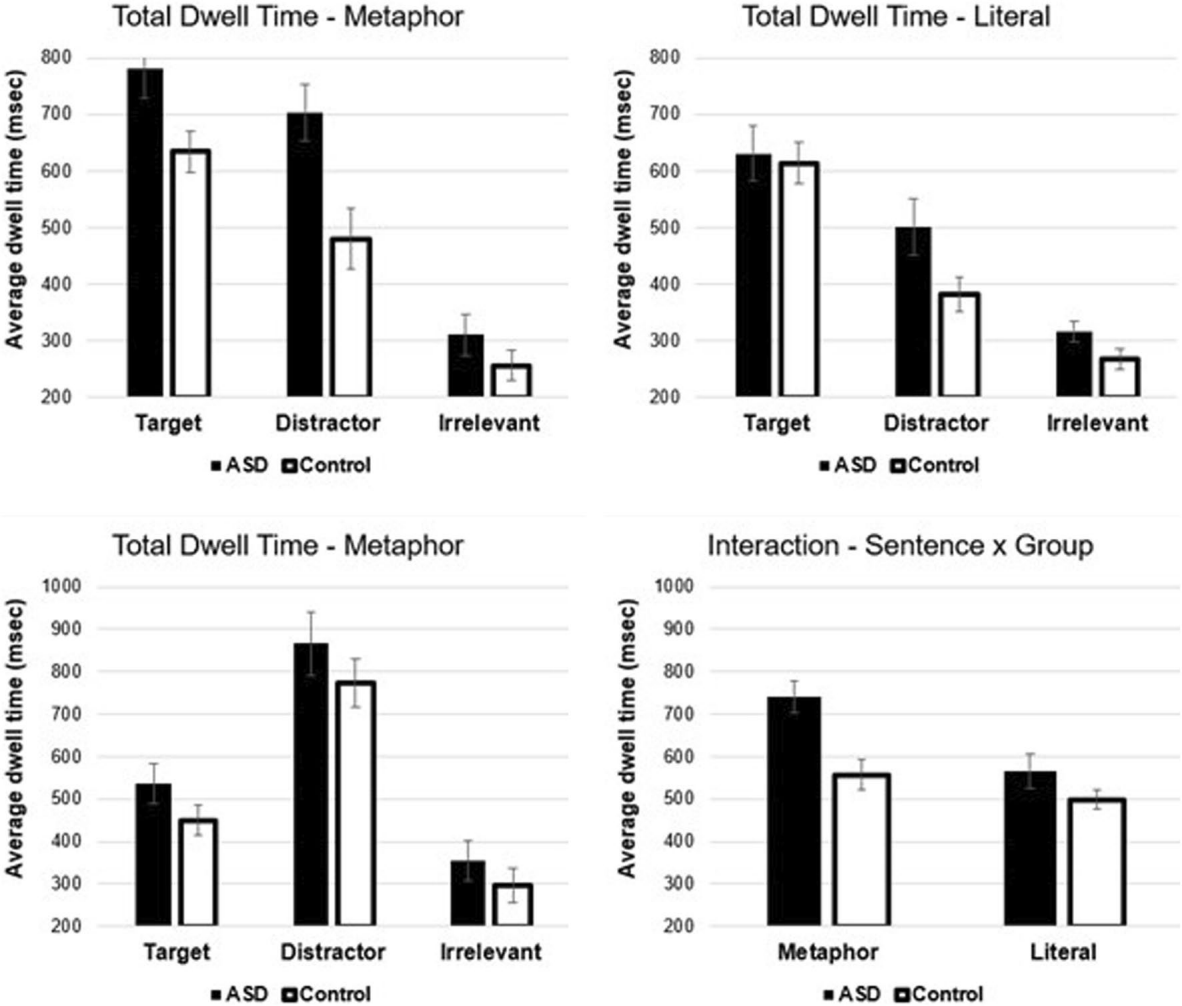
Mean fixation times. Upper panels show mean dwell times for the correct trials (left metaphorical and right literal). Lower left panel shows the mean dwell times for metaphor incorrect trials. Lower right panel shows the means for the sentence type by group interaction. Error bars show the standard error of the mean

*Region 2.* In the second time window, results showed significant main effects of picture type *F*(1,38) = 20.67, *p* < 0.001, *η*^*2*^ = 0.35, sentence type *F*(1,38) = 24.47, *p* < 0.001, *η*^*2*^ = 0.39, and group *F*(1,38) = 9.02, *p* < 0.01, *η*^*2*^ = 0.19 (see Fig. 4). Participants spent (1) more time viewing the target compared to the distractor, (2) more time fixating in metaphor trials compared to literal trials, and (3) participants with ASD had longer viewing times compared to controls. The latter two main effects are consistent with the reaction time analyses.

There was one significant two-way interaction between sentence type and group *F*(1,38) = 6.03, *p* < 0.05, *η*^*2*^ = 0.14 (see Fig. 4). Significant paired comparisons were observed between ASD and controls for metaphor trials *t*(38) = 3.55, *p* < 0.001, Cohen’s *D* = 1.13, and between metaphor and literal trials in participants with ASD *t*(17) = 5.15, *p* < 0.001, Cohen’s *D* = 1.21. The other two paired comparisons were not significant (literal-ASD vs. literal-control: *t*(38) = 1.54, *p* = 0.13, Cohen’s *D* = 0.49, and metaphor-control vs. literal-control: *t*(21) = 1.81, *p* = 0.08, Cohen’s *D* = 0.39). The interaction between sentence type and group is driven by the fact that individuals with ASD spent longer viewing the target and distractor in metaphor trials. Analysis of metaphor incorrect trials showed only a significant main effect of picture type *F*(1,38) = 41.82, *p* < 0.001, *η*^*2*^ = 0.52, which shows that the distractor was viewed longer than was the target (see Fig. 4). The main effect of group and interactions were not significant.

### Demographic and Vocabulary Analyses

The correlations between variables are presented in Table 2. Results showed that age significantly correlated with comprehension accuracy for metaphorical trials. Gender significantly correlated with dwell times to the distractor image on metaphor trials, and marginally correlated (1) with reaction times on metaphor trials and (2) dwell times to the target image on literal trials. In general, the pattern of correlations for the AQ scores largely mirrored the categorical “group” variable. Finally, vocabulary scores did not correlate with any of the key dependent measures.

**Table 2:** Bivariate correlations between demographic variables, diagnostic group, vocabulary, and metaphor processing task

Variable	1	2	3	4	5	6	7	8	9	10	11	12	13
1. Age	–	0.24	0.01	0.07	0.07	– 0.02	0.36*	– 0.04	– 0.02	– 0.21	0.07	– 0.01	– 0.10
2. Gender		–	– 0.33*	0.24	0.32^#^	0.19	0.06	0.22	0.31^#^	– 0.05	0.33*	0.30^#^	0.11
3. Group (ASD/TD)			–	– 0.79**	– 0.08	– 0.04	0.18	– 0.42**	– 0.52**	– 0.31^#^	– 0.44**	– 0.05	– 0.36*
4. AQ Total				–	0.15	– 0.06	– 0.18	0.47**	0.52**	0.29^#^	0.32*	0.10	0.32*
5. Vocabulary					–	– 0.02	– 0.11	– 0.17	– 0.20	– 0.01	0.05	0.13	– 0.02
6. Comp. Literal						–	– 0.26	0.32*	0.25	– 0.01	0.22	0.59**	0.34*
7. Comp. Metaphorical							–	– 0.02	– 0.11	– 0.25	– 0.17	– 0.13	– 0.04
8. RT Literal (correct)								–	0.85**	0.42**	0.45**	0.53**	0.45**
9. RT Metaphorical (correct)									–	0.25	0.63**	0.45**	0.44**
10. DW Distractor Literal R2										–	0.44**	0.11	0.15
11. DW Distractor Metaphor R2											–	0.33*	0.30^#^
12. DW Target Literal R2												–	0.42**
13. DW Target Metaphor R2													–

^*#*^*p* < 0.10, *p < 0.05, **p < 0.01. ASD coded 0 = ASD and 1 = control,* comp.*.  comprehension accuracy,* RT * reaction time,* DW*  dwell time

We conducted several backwards regression analyses in order to investigate whether demographic variables (age and gender) and/or vocabulary contributed significant variance above and beyond the group variable (ASD vs. TD control). In particular, we focused on metaphor trials and examined comprehension, reaction times for correct trials, and dwell times for correct trials (see Table 3). The results of the regression analyses showed that age was a significant predictor of comprehension, suggesting that older participants were more likely to select the metaphorical interpretation on metaphor trials. Second, gender was not a significant predictor in any of the regression analyses. Thus, the correlations, which showed that gender marginally correlated with metaphor reaction time and significantly correlated with distractor dwell time, did not survive (as significant) once group was included in the regression model.

**Table 3:** Backwards regression results and coefficients for predictor variables.

Variable	*B*	SE (B)	*β*	*t-*value (*p*-value)
*Metaphor comprehension F*(1,35) = 4.84, *p* < 0.05, *R*^*2*^ = 0.12				
Age	0.028	0.013	0.349	2.20 (0.034)
*Metaphor RT (correct) F*(2,34) = 8.41, *p* < 0.05, *R*^*2*^ = 0.33				
Group	− 1587.76	407,365	– 0.55	− 3.90 (< 0.001)
Vocabulary	− 43.06	24.96	– 0.24	− 173 (0.094)
*Metaphor DW target F*(1,35) = 5.62, *p* < 0.05, *R*^*2*^ = 0.14				
Group	− 153.63	64.79	– 0.37	− 2.37 (0.023)
*Metaphor DW distractor F*(1,35) = 7.01, *p* < 0.05, *R*^*2*^ = 0.17				
Group	− 211.20	79.76	– 0.41	− 2.65 (0.012)

To take the analysis of gender one-step further, we conducted partial correlations. The correlation between group and metaphor reaction time was − 0.52, and it was − 0.47 with gender partialled. Both of these correlations were significant (*p* < 0.05). Likewise, the correlation between group and metaphor distractor dwell time was − 0.44, and it was − 0.34 with gender partialled. Both of these were also significant (*p* < 0.05). For both partial correlations, there was some reduction in the correlation once gender variance was removed, but the remaining group effects were robust, and significant in both cases.

There was one marginal effect of vocabulary on reaction time in metaphor trials. As predicted, higher vocabulary scores were associated with lower reaction time. However, in general, there were not significant correlations between vocabulary and the main dependent measures on metaphor trials. The fact that vocabulary was retained in the regression analysis suggests that it accounts for unique variance over and above that accounted for by group, but given, that it was only marginally significant, caution is warranted in interpreting it.

## Discussion

The purpose of this study was to investigate novel metaphor processing in ASD. We hypothesized that individuals with ASD may not be significantly less likely than controls to choose the metaphorical meaning of critical utterances (e.g. Attwood, 1997; MacKay & Shaw, 2004; Rapin & Dunn, 2003; Tager-Flusberg, 2003), given that the evidence for impaired novel metaphor processing in ASD is mixed (Gold et al., 2010; cf. Giora et al., 2012; Hermann et al., 2013; Kasirer & Mishal, 2014, 2016; Mashal & Kasirer, 2011). We did expect that reaction times for metaphorical trials, in which the metaphorical meaning was chosen would be significantly slower in ASD. A key rationale for this study concerned online processing and what the eye movements may reveal in a Visual World Paradigm study (Tanenhaus et al., 1995). Specifically, we were interested in the extent to which individuals with ASD viewed the images corresponding to the literal and metaphorical interpretations, and whether these fixations mirrored performance in the control group or were distinct (Vulchanova et al., 2019). Specifically, eye movement should reveal the extent to which individuals with ASD consider the target image in metaphor trials.

Consistent with our hypotheses, comprehension showed a non-significant difference between groups, indicating that adults with ASD were not impaired in novel metaphor comprehension. That is, they interpreted the metaphor utterances, as metaphors, at the same rate as did the control participants (Giora et al., 2012; Hermann et al., 2013; cf. Chahboun et al., 2017; Mashal & Kasirer, 2011). Also, consistent with our hypotheses, reaction time analyses showed that participants with ASD took significantly longer to process metaphors. They were also slower on literal utterances. However, a base-line correction, which accounted for the additional processing time on literal trials, showed that it took participants with ASD almost 800 ms longer to process metaphor utterances as metaphors, compared to controls. The effect size (Cohen’s D) for this particular analysis was 1.05, indicating a large effect size. There are two similar reaction time results in the literature.

Gold and Faust (2010) reported an almost identical reaction time difference between controls (1003 ms) and ASD (1807 ms), when metaphorical expressions were presented to the left visual field, consistent with those authors assertions that the right hemisphere has a greater role in novel metaphor comprehension, due to semantic processing. However, it is important to bear in mind that the task used in the Gold and Faust study was quite different to the one used here. In contrast, Chahboun et al. (2017) examined reaction times in a novel metaphor comprehension task, and they reported a mean difference of approximately 875 ms between adults with ASD and typically-developing controls. However, in this study, there were no literal control trials, in which to baseline correct reaction time differences.

The additional time required for processing novel metaphors has obvious effects on individuals with ASD across a wide range of language comprehension situations (Olofson et al., 2014), and likely poses a barrier to successful communication across a range of contexts, which Gold and Faust (2010) refer to as “everyday soaked-in-metaphors in linguistic interactions” (p. 62). In text comprehension, where the reader controls the rate of input, it would lead to substantially slower overall reading times. The problem of slow metaphor processing would be compounded in interactive dialogue, which typically proceeds at a rate of 3–4 words a second, with little-to-no time between turns. Thus, by the time a metaphor is fully comprehended, the conversation will, in many cases, have moved on.

Recall that the filler trials contained idioms, which are another type of figurative language but conventional. We also analysed the comprehension and reaction times for idiom trials and results showed that participants with ASD had a mean of 2797 ms for correct trials and controls had a mean of 1933 ms (see Supplementary Materials, Section E). Thus, the mean reaction time for idioms for participants with ASD fell virtually in the middle between metaphor and literal trials, whereas for controls, idiom reaction time was just slightly higher than the metaphor trials. Thus, controls reaction times were nearly the same for both types of figurative language. Importantly, the difference score between metaphor and idiom for ASD was 567 ms and for controls was − 16 ms. This difference was statistically significant *t*(38) = 2.14, *p* < 0.05, Cohen’s *D* = 0.68, and again, shows that novel metaphor comprehension in ASD has a substantial processing cost implication compared to a distinct type of conventional figurative language (i.e. idioms). Whereas, controls did not show a difference between the two types of figurative language, and were, in general, substantially faster at processing both. Thus, the novel mapping between the target and vehicle seems to require significantly more time in individuals with ASD.

For eye movements, we observed two key findings. The first was that participants with ASD showed elevated target fixations in Region 1 (NP1), which at first glance should be a benefit to overall comprehension (i.e. give a head start on processing). However, after examining the fixations on incorrect trials, we instead interpret this finding as showing more uncertainty or slower visual information processing (Vulchanova et al., 2015), but at present, this conclusion is speculative. What we do know from our results is that there is a clear group difference in which participants with ASD showed approximately equal fixations to the target and distractor images during and shortly after NP1, whereas control participants showed significantly more fixations to the distractor image and fewer fixations on the target.

The second key eye movement finding is that participants with ASD showed elevated fixation times on both target and the distractor pictures in metaphor trials compared to controls, which we interpreted as a difficulty overcoming/suppressing the literal interpretation of metaphor utterances (Giora et al., 2012; Rubio Fernandez, 2007). The effect size of the key paired comparison here was 1.13, indicating a large effect size. However, we should note that Vulchanova et al. (2019), based on similar eye movement results, argued for greater uncertainty and/or competition between alternative interpretations (i.e. literal vs. metaphorical).^6^ We do not view these differences in interpretation as directly conflicting, but simply different ways of describing the same effect (or the same processing difficulty).

In cases where participants adopted a literal interpretation of a metaphorical sentence (i.e. incorrect metaphor trials), it was clear that participants almost solely focused on the distractor image and did not (or rarely) fixated the target image. Dwell times for incorrect trials showed a significant main effect of picture type (with a large effect size 0.52), and there was no difference between groups. Thus, both groups interpreted metaphor trials literally, at the same rate, and viewing behaviour did not differ between groups.

We included a section in the results, in which we consider two demographic variables and vocabulary scores. In an earlier study that focused on metaphor comprehension in dyslexia, we also did not find that vocabulary scores correlated with metaphor comprehension (*r* =  − 0.13) or metaphor reaction time (*r* =  − 0.04) (Cersosimo et al., 2024). The direction of the comprehension effect was not in the expected direction (i.e. higher vocabulary scores corresponded with worse comprehension). In the current study, we observed an almost identical correlation for comprehension (*r* =  − 0.11), whereas the reaction time was much higher (*r* =  − 0.20). Moreover, when vocabulary was included in a regression model with group (ASD vs. control), it was actually retained, suggesting that it had a significant impact on the *R*^2^, despite being only marginally significant (*p* = 0.09). Again, the direction of the effect on reaction times was in the expected direction, with higher vocabulary scores being negatively related to reaction time. One final point worth mentioning is that the correlations between AQ scores and comprehension, reaction time, and eye movements showed very similar results, as compared to the ASD group variable. Thus, treating ASD traits as a continuous variable did not account for further variance in the main dependent measures as compared to analysing ASD status categorically. In general, linear variables have more power potential compared to categorical variables.

To summarize, elevated fixation times on the distractor (in metaphor trials) suggests a difficulty suppressing the literal interpretation. This bias is despite more time viewing the target in the earlier time window, which is similar to the viewing pattern in incorrect trials. Although it is not entirely clear why an initial bias toward the target image in metaphor trials would lead to a greater tendency to interpret metaphorical utterances literally. Our speculation was that it was due to integration issues and/or slower visual processing, which is consistent with multi-modal integration arguments by Vulchanova et al. (2015). (Our task required integration of an auditory utterance with distinct visual representations.) Our integration speculation is also consistent with an increased tendency to fixate the distractor image in literal trials for participants with ASD (see upper left panel, Fig. 4). Importantly, in the literal condition, the distractor is an unrelated image, which clearly indicates some uncertainty on the part of participants with ASD. It is important to note that the distractor image is also the most complex image in the array, and so, a second possibility is that it receives additional fixation time (by both controls and participants with ASD) due to being more complex. Either way, the target image is fixated much longer in the key region for literal trials and the complexity issue does not substantially impact comprehension. For incorrect metaphor trials, neither group considers the target image, and viewing behaviour is almost exclusively focused on the distractor image.

### Limitations

The most important limitation of the study was the gender imbalance between groups (groups were matched on age and vocabulary). The ASD group was much more gender balanced compared to the controls, who were primarily female (~ 73%). Moreover, there were some gender differences in performance on the metaphor task, such that females were generally faster processors. We think this limitation is much less relevant given the results of the partial correlations and the regression analyses. When both group and gender were included in statistical models, ASD was a significant predictor and gender was not. We also considered dropping two or three females from the control group, which would have made the gender difference between groups not significant. However, this does not seem like good practice to us, and thus, we preferred to address the gender imbalance statistically. The results of those analyses confirm, that despite small-to-medium gender effects on reaction times, ASD status is much stronger and always survived analyses which also took gender into account. A second limitation of the current study is that it did not include a Theory of Mind test, which may have also accounted for unique variance in the dependent measures.

### Conclusions

This study has shown that adults with ASD comprehend novel metaphors at the same rate as controls, but they were substantially slower in doing so. Based on the comprehension results, we might conclude that individuals with ASD are not impaired at comprehending this particular type of figurative language, consistent with results from ASD adolescents (Kasirer & Mishal, 2016). But, what does the additional ~ 800 ms of processing time tell us? If we conclude that they are not impaired, then we at least have to conclude that they are less efficient processing novel metaphors. Some researchers have linked processing time of metaphors with cognitive issues due to executive functioning (e.g. Chiappe & Chiappe, 2007; Dietrich, 2004; cf. Russell, 1997), but given our data, that inefficiency is unlikely due to verbal abilities (see also Chahboun et al., 2017; Vluchanova et al., 2019). Furthermore, because we tracked eye movements, we are in a position to say that a good deal of the inefficiency is due to increased time spent fixating the distractor. The higher fixation time on the distractor clearly suggests some inability to switch or suppress the literal interpretation compared to typically-developing controls (Giora et al., 2012), who showed quite different patterns of viewing behaviour.

In terms of future directions, we believe that it will be important to assess novel metaphor comprehension alongside individual differences in inhibitory control. This will clarify whether a reader or listener needs to inhibit the literal interpretation to more efficiently process the metaphorical interpretation. A second important future direction is to examine metaphor processing in context, and specifically, moving towards more naturalistic conversation. It may very well be the case that processing metaphorical meaning(s) in context is where Theory of Mind becomes more of a critical issue, and therefore, present even more difficulties for individuals with ASD.

## Supplementary Information

Below is the link to the electronic supplementary material.

Supplementary file 1 (DOCX 474 kb)

ý

## Data Availability

The authors will make the data available following publication.

## Footnotes

1 One participant with ASD was excluded due to problems with eye tracking calibration.

2 Two additional windows were also considered and those results are presented in the Supplementary Materials (Sections B and C). The first was from the onset of the trial to the onset of NP1, and the second was from the onset of NP2 to the offset of NP2.

3 Participants in the study also completed an unrelated linguistic prediction task.

4 We also conducted one-sample *t-*tests on comprehension accuracy. That data is presented in the Supplementary Materials, Section A.

5 Literal trials had few comprehension errors, and hence, greater variability as revealed by larger error bars.

6 Mean fixation time differences between ASD and control were 400 ms on target-metaphor images and 200 ms on distractor-literal images.
