# Supplementary material for: Online Metaphor Comprehension in Adults with Autism Spectrum Disorders: An Eye Tracking Study
Source: J Autism Dev Disord. 2024 Sep 21;56(1):56–70. doi: 10.1007/s10803-024-06562-5 (PMC12860833; doi:10.1007/s10803-024-06562-5)
Supplement: Supplementary file 1 — (DOCX 474 kb) [file 10803_2024_6562_MOESM1_ESM.docx]

**Supplementary Materials**

**Section A: One-Sample *T*-Test Results**

We conducted one-sample *t*-tests to determine whether the comprehension means were significantly different than .50 (see upper left panel, Figure 2), which would indicate chance performance (omitting the irrelevant image). The literal conditions were significantly greater than .50 (ASD: *t*(17) = 16.44, *p* < .001, Cohen’s D = 3.87; control: *t*(21) = 16.70, *p* < .001, Cohen’s D = 3.56). The ASD group was not significantly different from chance for metaphor trials *t*(17) = .20, *p* = .85, Cohen’s *D* = .05, and the control group was significantly greater than chance for metaphor trials *t*(21) = 2.20, *p* < .05, Cohen’s *D* = .47.

**Section B: Initial Region Analysis**

The initial region of analysis was the time from when the picture appeared to the onset of NP1. In this window, there was only a significant main effect of picture type *F*(1,38) = 7.85, *p* < .01, *η^2^* = .17 (see Figure A). The distractor had increased fixation time compared to the target. The other main effects and interactions were not significant.


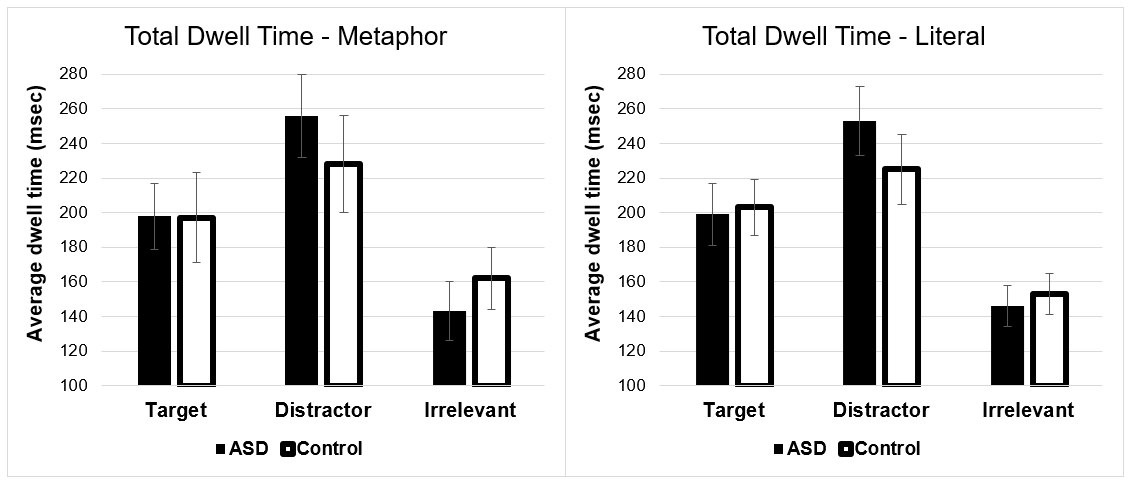


*Figure A.* Mean fixation times. Left panel shows mean dwell times for the metaphor trials, and the right panel shows the mean dwell times for literal trials. Error bars shows standard error of the mean.

**Section C: NP2 Analysis**

Figure B shows the fixation data from the onset of NP2 to the offset of NP2 (see also Figure 1). In this window, there was a significant main effect of picture type *F*(1,38) = 4.20, *p* = .05, *η^2^* = .10 The target had increased fixation time compared to the distractor. There was also an interaction between sentence type and picture type *F*(1,38) = 7.45, *p* = .01, *η^2^* = .16. This interaction is driven by the fact that there are more looks to the target and less looks to the distractor in literal trials *t*(39) = -4.37, *p* < .001, Cohen’s *D* = -.69, whereas in the metaphor trials, fixations were more-or-less equally distributed between the target and distractor *t*(39) = .58, *p* = .57, Cohen’s *D* = .09. In addition, there were more looks to the target in literal trials compared to metaphor trials *t*(39) = 3.13, *p* < .05, Cohen’s *D* = .50, and fewer looks to the distractor in literal trials compared to metaphor trials *t*(39) = -2.13, *p* < .05, Cohen’s *D* = -.34. The other main effects and interactions were not significant. Finally, we considered fixation times for incorrect metaphor trials (see Figure B). Results showed only a marginally significant main effect of picture *F*(1,38) = 4.03, *p* = .052, *η^2^* = .10. There were more fixations on the distractor compared to the target. The main effect of group and the interaction between picture and group were not significant.


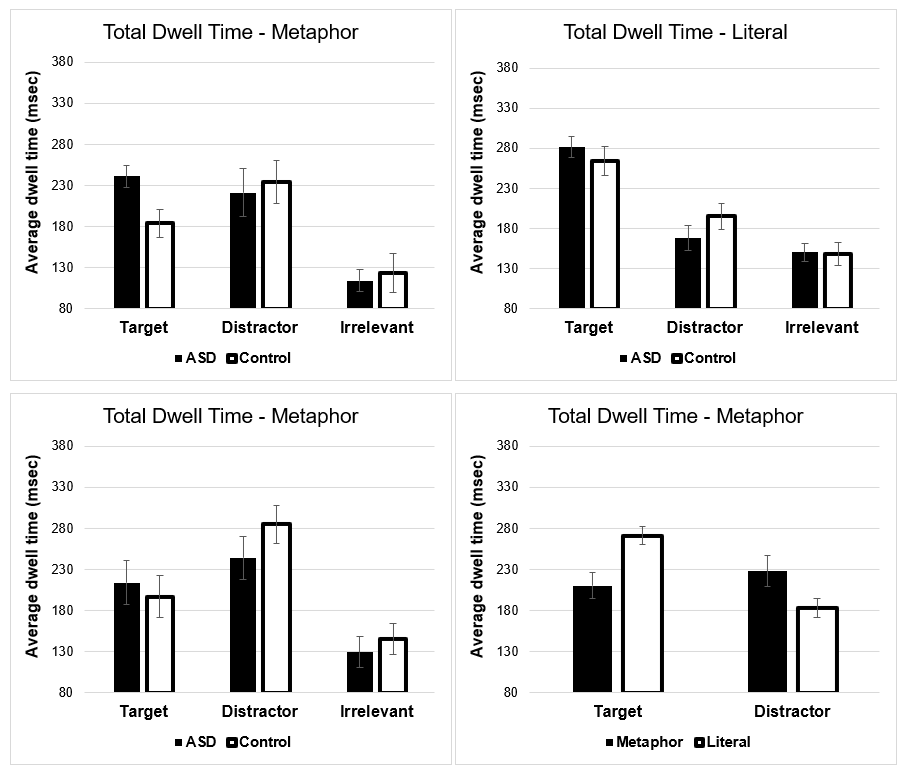


*Figure B.* Mean fixation times. Top left panel shows mean dwell times for the metaphor trials, and the top right panel shows the mean dwell times for literal trials. The bottom left panel shows dwell times for incorrect trials. The bottom right panel shows the picture type by sentence type interaction. Error bars shows standard error of the mean.

**Section D: Outlier Analysis**

There was one outlier in reaction times (*Z* scores: 3.57-4.06 SDs). That participants reaction time was elevated for both literal and metaphorical trials, and was similar between the “all trial” and “correct trial” analyses. Importantly, that participant did not have outlying scores for either comprehension or fixation times (all *Z’s* < 3.0 SDs). In addition, this participant did not have elevated idiom reaction times.

When the outlying participants’ data was removed, results for all trials showed significant main effects of sentence type *F*(1,37) = 38.60, *p* < .001, *η^2^* = .51, in which literal sentences were processed more quickly compared to metaphor sentences, and group *F*(1,37) = 12.25, *p* = .001, *η^2^* = .25, where controls had shorter reaction times than did the ASD group (see Figure C). The interaction was also significant *F*(1,37) = 7.72, *p* < .01, *η^2^* = .17. Reaction times for correct trials confirmed significant main effects of sentence type *F*(1,37) = 22.97, *p* < .001, *η^2^* = .38 and group *F*(1,37) = 14.50, *p <* .001, *η^2^* = .28, and a significant interaction between variables *F*(1,37) = 9.17, *p* < .01, *η^2^* = .20 (see Figure C). For both ANOVAs, the main effect of group was numerically larger with the outlier removed. Thus, the outlier had no effect on inferential analyses.


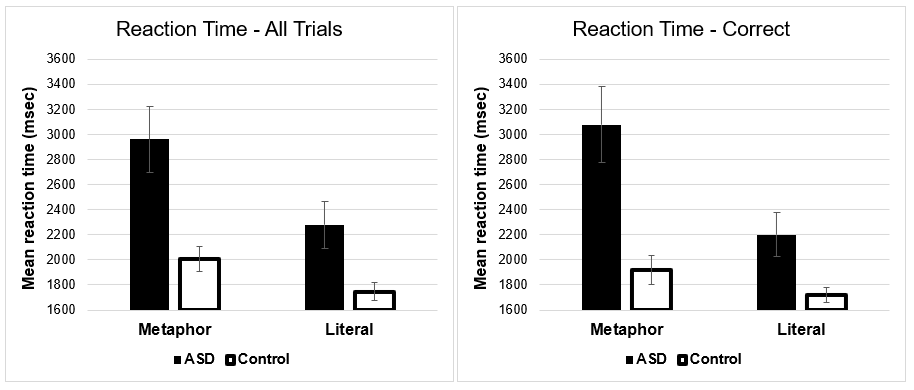


*Figure C.* Mean reaction times for all trials and correct trials, with outlier removed. Error bars show the standard error of the mean.

**Section E: Idiom Results**

For comprehension, the ASD group was not significantly different controls *t*(38) = -.24, *p* = .82, Cohen’s *D* = -.08 (see Figure D and E). Furthermore, neither group was significantly different from chance .50 (ASD: *t*(17) = -.55, *p* = .59, Cohen’s *D* = -.13, and controls: *t*(21) = .47, *p* = .93, Cohen’s *D* = -.02. For reaction times, the ASD group was significantly different from controls for both correct trials *t*(38) = 3.44, *p* < .001, Cohen’s *D* = 1.09 and all trials *t*(38) = 3.77, *p* < .001, Cohen’s *D* = 1.20.


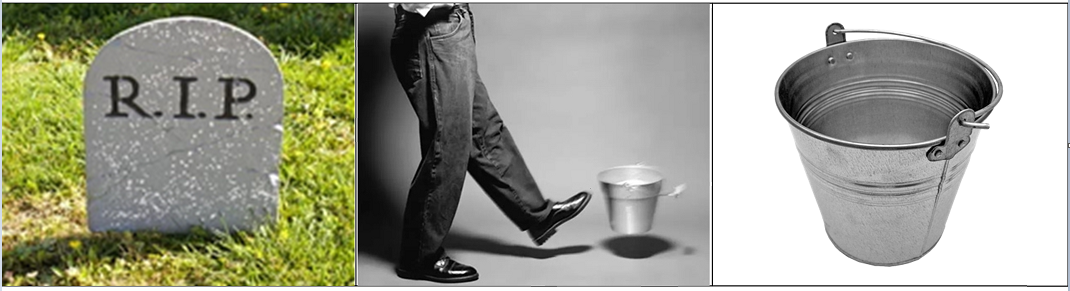


*Figure D.* Example item for idiom trial. The corresponding utterance was *He kicked the bucket.*


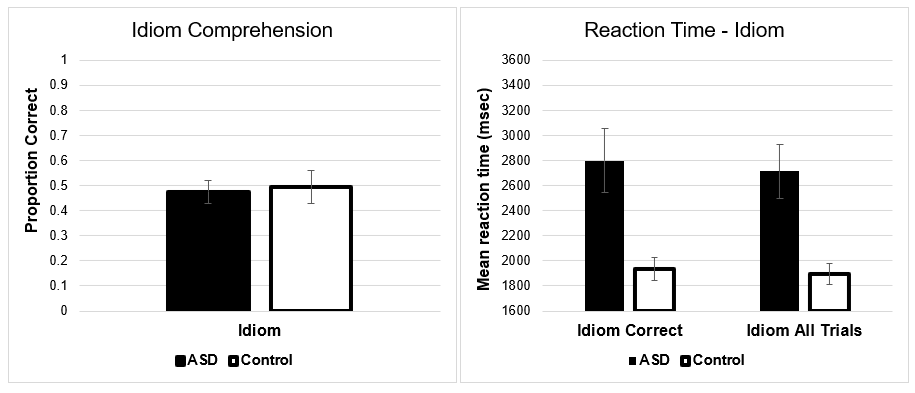


*Figure E.* Proportion correct and mean reaction times for idiom trials. Error bars show the standard error of the mean.
